# The therapeutic strategy for advanced gastric cancer with pyloric stenosis and liver metastasis; successfully treated by gastro-jejunal bypass and chemotherapy first, followed by curative R0 resection

**DOI:** 10.1186/s40792-020-00979-7

**Published:** 2021-01-06

**Authors:** Naosuke Nakamichi, Masahiro Tsujiura, Tomohiro Matsui, Taiga Yamamoto, Ayana Yoshioka, Hidekazu Hiramoto, Yoshimi Ouchi, Takeshi Ishimoto, Toshiyuki Kosuga, Satoshi Mochizuki, Susumu Nakashima, Masamichi Bamba, Mamoru Masuyama, Eigo Otsuji

**Affiliations:** 1 Department of Surgery, Saiseikai Shiga Hospital, 2-4-1 Ohashi, Ritto, Shiga 520-3046 Japan; 2 Department of Diagnostic Pathology, Saiseikai Shiga Hospital, 2-4-1 Ohashi, Ritto, Shiga 520-3046 Japan; 3 Division of Digestive Surgery, Department of Surgery, Kyoto Prefectural University of Medicine, 465 Kajii-cho, Kawaramachihirokoji, Kamigyo-ku, Kyoto, Kyoto 602-8566 Japan

**Keywords:** Gastric cancer, Liver metastasis, Preoperative chemotherapy, Pyloric stenosis, Gastro-jejunal bypass

## Abstract

**Background:**

The indication of surgical resection for liver metastasis from gastric cancer (GC) is still limited and controversial because of its more aggressive oncological characteristics than liver metastasis from colorectal cancer. Pyloric stenosis causes an inadequate oral intake and malnutrition in GC patients. We herein report a case of GC with these two factors that was successfully treated by the combination of gastro-jejunal bypass and chemotherapy, followed by curative R0 resection.

**Case presentation:**

A 60-year-old man was diagnosed with type 2 GC with liver metastasis and pyloric stenosis, which was confirmed as the HER2-positive type. He underwent gastrojejunostomy and received capecitabine and cisplatin (XP) + trastuzumab chemotherapy. After three courses of the XP + trastuzumab regimen, shrinkage of the primary lesion and liver metastasis was confirmed and his nutritional parameters markedly improved with a stable oral intake after bypass surgery. He underwent curative R0 resection by distal gastrectomy with D2 lymphadenectomy and partial hepatectomy. Histologically, viable tumor cells were observed in less than one-third of the primary lesion, and only scar tissue without viable cancer cells was noted in the resected liver specimen. His postoperative course was uneventful, and recurrence has not been detected in the 30 months after surgery without adjuvant chemotherapy.

**Conclusion:**

The present case report describes a successful strategy for advanced GC with pyloric stenosis and liver metastasis.

## Background

Although chemotherapy is regarded as the standard treatment for recurrent and metastatic gastric cancer (GC) [1], hepatectomy for liver metastasis is a treatment option aimed at macroscopic complete resection (R0 resection) [2, 3]. The indication of surgical resection for liver metastasis from GC remains limited and controversial [4] because of its more aggressive oncological characteristics than liver metastasis from colorectal cancer [5]. The combination of peri-operative chemotherapy and liver resection has been widely introduced as a multidisciplinary treatment for GC with liver metastasis; however, it currently remains unclear whether preoperative/neo-adjuvant or adjuvant chemotherapy is more beneficial for survival outcomes [6, 7].

Pyloric stenosis is an obstructive condition of the gastric outlet induced by tumor growth in the pyloric area and causes nutritional disorders. Preoperative malnutrition is recognized as a negative indicator of short- and long-term outcomes after curative gastrectomy for GC [8, 9]. Furthermore, GC patients with pyloric stenosis have worse survival outcomes than those without pyloric stenosis, and this is mainly due to higher rates of deep tumor invasion and lymph-node metastasis [10].

We herein report a case of advanced GC with liver metastasis and pyloric stenosis that was successfully treated by the combination of gastro-jejunal bypass and chemotherapy, followed by curative R0 resection. This therapeutic strategy is a treatment option for advanced GC patients with these two factors.

## Case presentation

**Fig. 1:**
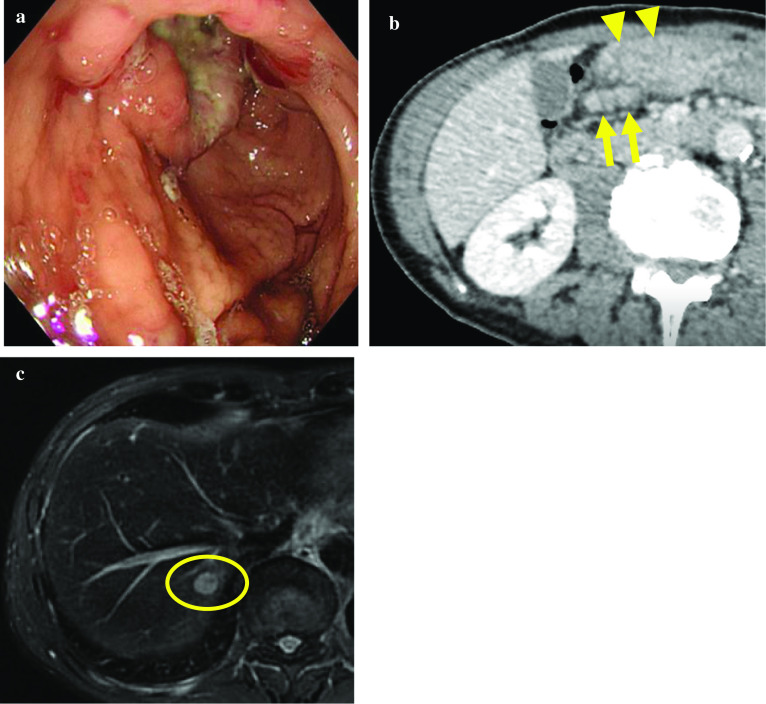
Image findings at the initial hospital visit (before any treatment). **a** Upper gastrointestinal endoscopy showed a type 2 circumferential tumor with a gastric outlet obstruction located at the gastric antrum. **b** Abdominal contrast-enhanced CT revealed a thickened gastric wall at the pre-pyloric area (triangle) and two swollen regional lymph nodes at station No. 6 (arrow). **c** On MRI, a highly enhanced lesion measuring 10 mm in diameter was detected in the liver S7 (circle)

A 60-year-old man visited our hospital with bloody vomiting. Upper gastrointestinal endoscopy showed a circumferential ulcerative lesion measuring 40 mm at the pre-pyloric area, which caused tumor bleeding and pyloric stenosis (Fig. 1a). Biopsy revealed well-differentiated to poorly differentiated adenocarcinoma (tub1/por), which was later confirmed to be the HER2-positive type. A blood examination showed severe anemia (hemoglobin level: 5.5 g/dL) and an unfavorable nutritional status (total protein: 4.5 g/dL, albumin level: 2.5 g/dL, pre-albumin: 16.7 mg/dL). Regarding tumor markers, his AFP level was slightly elevated (53.6 ng/mL), while CEA and CA19-9 levels were within normal ranges (Table 1, left side). Contrast-enhanced abdominal CT showed thickening of the gastric wall at the pre-pyloric area (primary lesion) and two swollen regional lymph nodes (station No. 6) (Fig. 1b). MRI was performed, and a high-intensity lesion measuring 10 mm in diameter was identified in the liver (S7) on a T1-weighted image, suggesting solitary liver metastasis from GC (Fig. 1c). As pretreatment staging, he was diagnosed with advanced GC located at the lower third of the stomach that was 40 mm in size and macroscopically type 2, tub1/por, cT4a(SE)N1M1(HEP) cStage IV according to the third English edition of the Japanese classification of gastric carcinoma [11].

**Table 1:** Laboratory data before and after chemotherapy

	Normal range	Before chemotherapy	After chemotherapy
Total protein (g/dL)	6.1–8.2	4.5	6.7
Albumin (g/dL)	3.9–5.0	2.5	4.2
Pre-albumin (g/dL)	21.0–41.0	16.7	33.8
Hemoglobin (g/dL)	13.7–16.8	5.5	11.6
White blood cell count (/mm^3^)	3200–8500	4100	5100
Total lymphatic count (/mm^3^)	1100–3300	1312	1571
Prognostic nutritional index (PNI)^a^	≥ 40	31.6	49.9
AFP (ng/mL)	≤ 10.0	53.6	13.7
CEA (ng/mL)	≤ 5.0	4.4	4.5
CA19-9 (U/mL)	≤ 37.0	3.5	2.9

^a^PNI = (10 × albumin (g/dL)) + (0.005 × total lymphatic count (/mm^3^))

**Fig. 2:**
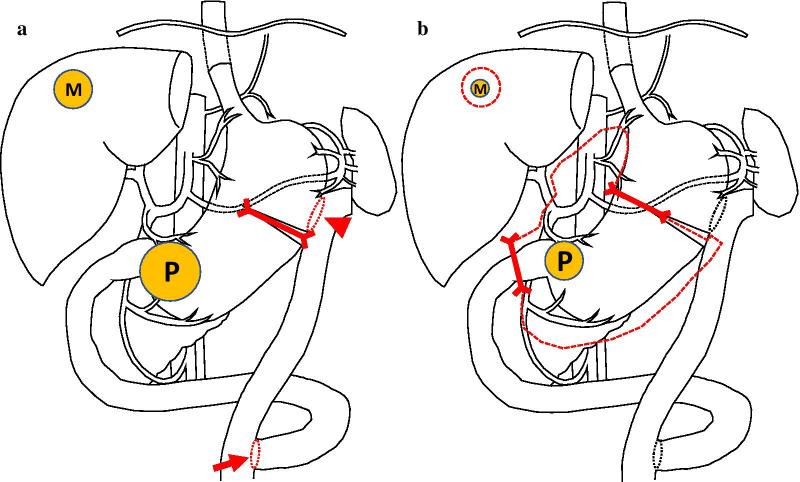
Schema of each surgery. **a** First surgery; gastro-jejunal bypass (Roux-en-Y gastric bypass) and modified Devine exclusion were performed. Gastrojejunostomy (triangle) and jejuno-jejunostomy (arrow). **b** Second surgery; distal gastrectomy with D2 lymphadenectomy and partial hepatectomy were performed (red dotted line). P; primary lesion, M; metastatic lesion of the liver, and Red thick line; resection line of the stomach and duodenum

The present case was diagnosed with advanced GC with potentially/technically resectable liver metastasis and a nutritional disorder due to pyloric stenosis. We considered two therapeutic options: (i) surgery (standard primary tumor resection with lymphadenectomy and liver metastasectomy) with/without adjuvant chemotherapy, and (ii) systemic chemotherapy (as a neo-adjuvant setting) followed by surgical resection. After sufficient discussions and the provision of informed consent from the patient and his family, we selected the latter therapeutic strategy. Before the introduction of systemic chemotherapy, we performed gastro-jejunal bypass (Roux-en-Y gastric bypass) with the laparoscopic approach to overcome the food obstruction issue. Since modified Devine exclusion was added to bypass surgery [12, 13], the greater curvature side of the gastric body was transected using a linear cutting stapler to partition the stomach wall (Fig. 2a). During laparoscopic surgery, we confirmed no findings of peritoneal metastasis.

**Fig. 3:**
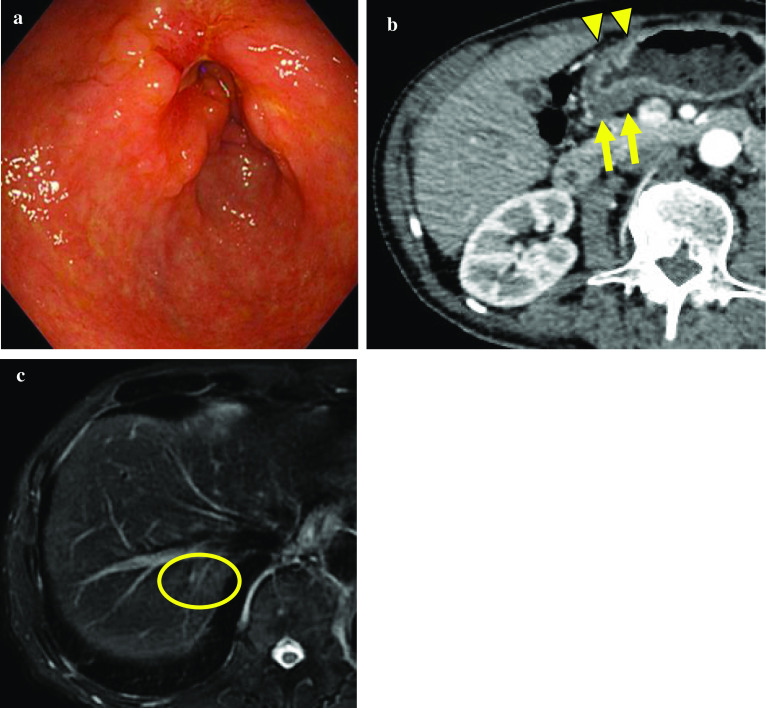
Image findings after preoperative chemotherapy. **a** Upper gastrointestinal endoscopy showed marked shrinkage of the primary tumor. **b** On CT, shrinkage of the primary tumor (triangle) and regional lymph nodes (arrow) was confirmed. **c** MRI showed marked shrinkage of liver metastasis (circle)

As a systemic chemotherapy, capecitabine and cisplatin in combination with intravenous trastuzumab (the XP plus T-mab regimen) were selected. The postoperative course after gastro-jejunal bypass was uneventful, and the patient received initial administration on the 9th day after surgery. The XP regimen comprised 1000 mg/m^2^ capecitabine (Xeloda^®^) orally twice a day on days 1–14 and 80 mg/m^2^ cisplatin intravenously on day 1, which was repeated every 3 weeks. An intravenous injection of 8 mg/kg trastuzumab was administered on day 1 of the first cycle and then at 6 mg/kg on day 1 of subsequent cycles. Although grade 3 neutropenia according to the National Cancer Institute Common Terminology Criteria for Adverse Events (NCI-CTCAE) [14] was observed before the induction of the second cycle, his overall therapeutic course was uneventful. After three courses of the XP plus T-mab regimen, upper gastrointestinal endoscopy, CT, and MRI were performed and showed shrinkage of the primary tumor and regional lymph nodes as well as the disappearance of liver metastasis (Fig. 3). Additionally, no newly detected metastatic lesion was found in the liver or other organs. The clinical stage following chemotherapy was ycT3(SS)N0M0, ycStage IIB.

Laboratory data after chemotherapy are summarized in Table 1 (right side), and showed improved nutritional data (total protein: 4.5–6.7 g/dL, albumin: 2.5–4.0 g/dL, pre-albumin: 16.7–33.8 mg/dL), and a decreased AFP level (53.6 to 13.7 ng/mL). Along with this improvement in the nutritional status, the prognostic nutritional index (PNI) for postoperative complication risk factors was also elevated (31.6 to 49.9) [15].

**Fig. 4:**
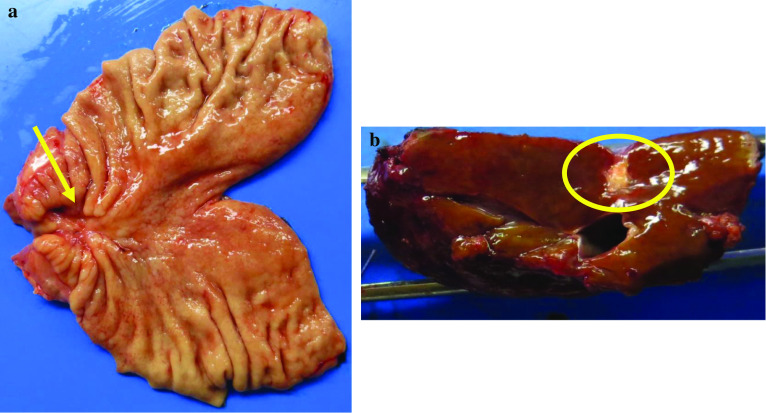
Macroscopic findings of resected organs. **a** Stomach; a type 2 lesion was detected macroscopically in the gastric antrum (red arrow). **b** Liver; a liver lesion was confirmed as scar tissue

We considered surgical curability and safety to be achievable at this point. The patient underwent macroscopic complete resection (R0 resection) by distal gastrectomy, D2 lymphadenectomy, and partial hepatectomy of S7. The previous transection line of modified Devine exclusion procedure was extended to the lesser curvature side in the second surgery (Fig. 2b). The hepatic lesion, which was recognized as a scarring lesion on the surface of the liver (S7), was resected with a sufficient safety margin (Fig. 4).

**Fig. 5:**
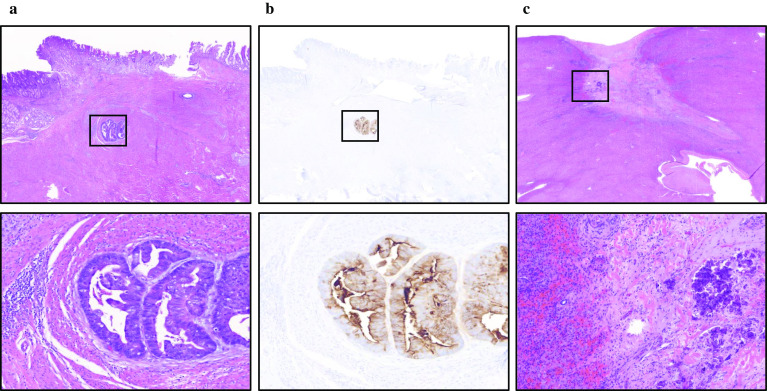
Microscopic findings of resected organs. **a** Stomach; Viable tumor cells were observed in less than one-third of the lesion and more than two-thirds were replaced with scar tissue. **b** AFP immunochemistry; AFP was focally stained in the primary lesion. **c** Liver; Only scar tissue without viable cancer cells was observed

Pathologically, the primary lesion was moderately differentiated adenocarcinoma (tub2) and invaded the subserosal layers (T3(SS)). Viable tumor cells were observed in less than one-third of the lesion and more than two-thirds were replaced with scar tissue (Fig. 5a), indicating a grade 2 tumor response by preoperative chemotherapy [11]. An immunohistochemistry of the resected primary lesion revealed focally positive for AFP (Fig. 5b). One lymphatic metastasis was observed in the subpyloric area (station No. 6) (N1). In the resected liver specimen, only scar tissue without viable cancer cells was observed (Fig. 5c). Collectively, these results indicated that the pathological staging was ypT3(SS)N1(1/30)M0, ypStage IIB.

**Fig. 6:**
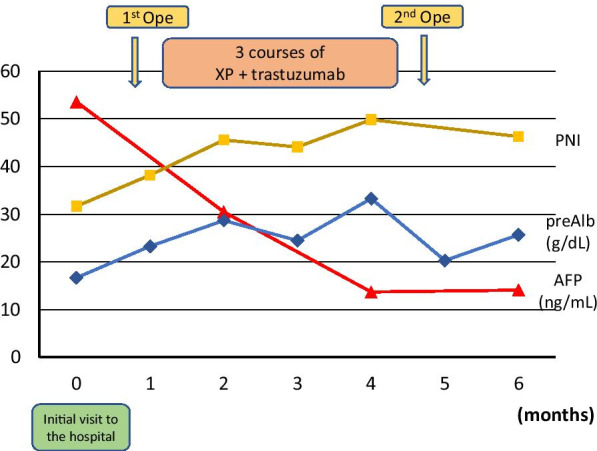
Clinical course of the present case. The clinical course and date since “initial visit to the hospital”. “First Ope” is gastro-jejunal bypass and “Second Ope” is distal gastrectomy with D2 lymphadenectomy and partial hepatectomy. Normal range; AFP (alpha-fetoprotein;) ≤ 10.0 (ng/mL), PNI (prognostic Nutritional Index) ≥ 40, and Pre-ALB (pre-albumin) 21.0–41.0 (g/dL)

The postoperative course was uneventful, and the patient was discharged from hospital on postoperative day 10. We proposed adjuvant chemotherapy; however, the patient and his family did not hope it. After enough consultation, we finally decided not to administer postoperative adjuvant chemotherapy. He was followed up every 3 months and has remained free from recurrence 30 months after surgical resection. The outline of the clinical course is summarized in Fig. 6.

## Discussion

The present case of GC had liver metastasis and pyloric stenosis. We preceded gastro-jejunal bypass and systemic chemotherapy for this case with obstruction of the food passage and clinically solitary liver metastasis. Chemotherapy was uneventfully administrated, and shrinkage of both the primary tumor and metastatic lesions was achieved. Furthermore, anemia, malnutrition, PNI score, and AFP level were all improved mainly by a stable oral intake after bypass surgery. We then safely performed curative R0 resection by distal gastrectomy, D2 lymphadenectomy, and partial hepatectomy.

GC with distant metastasis (M1) is defined as Stage IV regardless of any T or N factor [11]. Distant metastases of GC vary and include extra-regional lymph node, hematological, and peritoneal metastases. Regarding the strategy for all stage IV GC, Yoshida et al. classified stage IV GC into four categories according to the type and number of non-curative factors, and proposed the recommended treatment strategies for each category [16]. The present case was classified as “potentially resectable metastasis”, for which surgical resection or the combination of preoperative chemotherapy was recommended.

The indication of surgery for stage IV GC with liver metastasis is still not defined. Previous studies demonstrated that the prognosis of patients may be improved by careful selection for surgical indication, such as non-serosal invasion of the primary tumor, one or a few liver metastases, and smaller size of metastatic lesions [2–4, 17–19]. The Japanese Gastric Cancer Association guidelines suggest a multidisciplinary approach, including surgery with curative intent, when the number of liver metastatic nodules is small, provided that other non-curative factors are absent [20].

The present case was Stage IV GC with one liver metastasis of 1 cm in size, and was considered to be technically resectable. Although there were two options, surgery first or chemotherapy first, we selected the latter after sufficient consideration and informed consent. This was a very difficult decision to reach and was ultimately based on the need to (i) monitor liver metastasis over time and confirm whether liver metastasis was truly solitary, and (ii) improve his general and nutritional conditions prior to radical surgery. Since the present case was technically resectable, this preoperative chemotherapy was considered as a neo-adjuvant setting.

Recently, the long-term outcomes of the conversion therapy for GC have been reported by several institutes. Yamaguchi et al. treated 259 patients who had stage IV GC with chemotherapy and performed conversion surgery in 84 patients of them [21]. The median survival time of the patients who underwent surgical resection was 30.5 months (especially 41.3 months with R0 resection), as opposed to those who received chemotherapy alone at 11.3 months. Boen et al. retrospectively investigated oncologic outcomes for 101 patients with stage IV GC who were treated with systemic chemotherapy followed by gastrectomy [22]. In this report, compared with the prognoses for patients with other types of distant metastasis, the prognosis for patients with liver metastasis was better. Since the present case had stage IV GC with one liver metastasis, favorable long-term outcomes might be expected after R0 resection by curative gastrectomy plus metastasectomy.

The nutritional status has been reported to have a number of clinical impacts on the management of GC patients, including surgical intervention and peri-operative chemotherapy [23, 24]. Regarding surgical safety, preoperative PNI and pre-albumin levels have been correlated with postoperative complications after gastrectomy [25–28]. In terms of chemotherapy, a favorable nutritional condition has been associated with better clinical and survival outcomes in various malignant diseases, including GC [24, 29–31]. In the present case, the patient had an unfavorable nutritional status at the initial hospital visit because of an inadequate oral intake due to pyloric stenosis. After achieving a stable oral intake following bypass surgery, systemic chemotherapy could be administered uneventfully. His nutritional data and general condition also markedly improved after three cycles of chemotherapy. Accordingly, we could perform curative surgery without any postoperative complications. Migita et al. focused on the time course of PNI and calculated differences between prior to neo-adjuvant chemotherapy and before gastrectomy [32]. Interestingly, decreased PNI values were associated with a worse long-term outcome. Since the present case showed increased PNI value after chemotherapy, better long-term outcome might be expected for him.

The ToGA trial confirmed the efficacy of trastuzumab for inoperable locally advanced, recurrent, or metastatic HER-2-positive adenocarcinoma of the stomach or gastro-esophageal junction cancer [33]. Some case reports have demonstrated the usefulness of trastuzumab for GC patients as preoperative therapy, followed by radical gastrectomy [34–36]. The present case received the trastuzumab-included regimen, which exerted sufficient anti-tumor effects, as might be expected. Although adjuvant chemotherapy using the trastuzumab-included regimen has been conducted in some case reports [35, 36], there is still no evidence regarding postoperative therapy.

In the present case, we successfully administered effective preoperative chemotherapy and performed R0 resection by distal gastrectomy and partial hepatectomy. However, this treatment strategy has several disadvantages. The patient might miss the opportunity for surgery due to disease progression during chemotherapy. Since a consensus for preoperative (neo-adjuvant) chemotherapy for such a case has not yet been established, further evidence is needed in the near future. Another disadvantage is that the patient underwent surgery twice in the current treatment strategy. Although physical stress generally needs to be minimized, the first surgery was considered to be necessary to secure an oral intake and increase the safety of consequent radical resection. Other disadvantages include the possibility of the adverse effects of chemotherapy for surgical intervention (increased postoperative complications), a longer treatment period, and higher treatment costs.

## Conclusions

The present case report demonstrates a successful strategy for advanced GC with pyloric stenosis and liver metastasis.

## Data Availability

The data are not available for public access because of patient privacy concerns but are available from the corresponding author on reasonable request.

## Abbreviations

GC: Gastric cancer
PNI: Prognostic nutritional index
